# Combining Native and Malted Triticale Flours in Biscuits: Nutritional and Technological Implications

**DOI:** 10.3390/foods12183418

**Published:** 2023-09-13

**Authors:** Isabella Piazza, Paola Carnevali, Nadia Faccini, Marina Baronchelli, Valeria Terzi, Caterina Morcia, Roberta Ghizzoni, Vania Patrone, Lorenzo Morelli, Mariasole Cervini, Gianluca Giuberti

**Affiliations:** 1 Centre BIOGEST-SITEIA, Department of Life Science, University of Study of Modena and Reggio Emilia, Via Amendola, n. 2, 42122 Reggio Emilia, Italy; 2 R&D Food Microbiology & Molecular Biology Research, Barilla G. e R. Fratelli S.p.A., 43122 Parma, Italy; paola.carnevali@barilla.com; 3 Research Centre for Genomics and Bioinformatics, Council for Agricultural Research and Economics, 29017 Fiorenzuola d’Arda, Italy; nadia.faccini@crea.gov.it (N.F.); marina.baronchelli@crea.gov.it (M.B.); valeria.terzi@crea.gov.it (V.T.); caterina.morcia@crea.gov.it (C.M.); roberta.ghizzoni@crea.gov.it (R.G.); 4 Department for Sustainable Food Process (DiSTAS), Università Cattolica del Sacro Cuore, 29122 Piacenza, Italy; vania.patrone@unicatt.it (V.P.); lorenzo.morelli@unicatt.it (L.M.); mariasole.cervini@unicatt.it (M.C.); gianluca.giuberti@unicatt.it (G.G.)

**Keywords:** biscuits, malted triticale flour, dietary fiber, texture, in vitro digestion

## Abstract

Triticale-based biscuits were formulated with increasing substitution levels (i.e., 0, 25, 50, 75, and 100% *w*/*w*) of malted triticale flour (MTF). The products were analyzed for technological and nutritional characteristics, including the evaluation of the in vitro starch digestion. The results indicated that the substitution of triticale flour with MTF increased (*p* < 0.05) the total dietary fiber and ash contents. Total starch decreased (*p* < 0.05) when the level of MTF increased in the formulation, causing an increase in reducing sugars and an increase in the starch hydrolysis index and in the in vitro predicted glycemic index (pGI). The hardness and spread ratio values of biscuits decreased (*p* < 0.05) with increasing levels of MTF in the recipe. The lightness of doughs and biscuits decreased (*p* < 0.05) with increasing MTF levels. Overall, MTF could be used to formulate biscuits with higher dietary fiber content than native triticale flour and a medium to high in vitro glycemic index value as a function of the substitution level.

## 1. Introduction

Triticale (*Triticosecale wittmack*) is a wheat/rye hybrid grain with a worldwide production that has consistently increased during the last two decades, reaching about 17 million tonnes in 2014 [1]. Triticale was traditionally used as animal feed and for biofuel production; however, the growing demand for food resources and the current consumer trend of trying novel products has led to an increased interest in food production [2]. In addition, triticale could be an important crop to ensure food security due to its tolerance to drought, disease, more acid soils, low susceptibility to biotic stresses, and high grain yield even in marginal environments [3,4].

From a nutritional standpoint, the chemical composition of triticale is more similar to wheat than rye due to its genome proportions [5]. Accordingly, wheat, rye, and triticale flours contained similar total protein contents but different protein fractions and amino acid composition [6]. In addition, triticale total starch content (i.e., 63.3–68.8 g/100 g dry matter) is comparable to wheat and rye; however, the ratio of amylose to amylopectin can vary considerably [5]. Considering the dietary fiber content, triticale has a high amount of soluble fraction, especially water-extractable arabinoxylans [5,6].

Several studies have been conducted to formulate triticale-based foods in recent years. Most works were focused on the development of triticale flour dough suitable for breadmaking [7,8,9]. The results indicated that triticale was characterized by a low gluten quantity and quality, and triticale typically exhibits low falling number and lower dough stability and dough development time than wheat [7,8,9]. Contrarily, other studies reported that triticale flour was better suited for unleavened baked products, including biscuits and crackers [10,11]. For instance, an improvement in the spread ratio of biscuits made by blending triticale with wheat flour has been reported [10,11,12,13].

Other than the use of native flour, triticale is a promising cereal for malting and brewing owing to its high levels of α-amylase and proteolytic enzymes, which allow a short soaking time and a quick malting process [3]. During the malting process, which involves soaking, germination, and drying, several physicochemical changes can occur that can positively affect the grain’s chemical and nutritional composition regarding macro- and micro-nutrients and bioactive compounds [14]. Through the milling of the malted grains, malt flour is produced, which can often be added to wheat flour in adequate amounts to improve the technological and sensorial properties of bread [14]. In addition, previous studies indicated that malted sorghum flour can be used in place of up to 60% (*w/w*) of wheat flour for the preparation of nutritionally enhanced biscuits without changes in texture, crispiness, appearance, and overall acceptability [4,14].

Given the optimal nutritional value of malted flours, the good attitude of triticale for the malting process, the suitability of triticale flour for biscuits preparation, and the trend in the food market to formulate baked products with unconventional and under-exploited flours, this study aimes to formulate novel biscuits made only with triticale flour and malted triticale flour (MTF) in different ratios. To our knowledge, this is the first study in which triticale flour (native or malted) was exclusively employed in biscuit formulation. Exploring the potential of triticale flour as the base ingredient in biscuits could spawn consumer and stakeholder interest to seek out cereal-based products made from cereal grains other than common wheat cultivars. Indeed, different studies dealing with the formulation of biscuits produced from malted flours are currently present in the literature but using the following cereals: wheat, barley, buckwheat, oat, sorghum, and millet [4,10,11,14,15,16]. In most of these works, biscuits are made with composite flours containing malted and native flours from the same cereal and blended in different ratios with other grains (mainly wheat) and pulses. In this work, to better explore the suitability of triticale and MTF in biscuits, products were analyzed in terms of technological and nutritional attributes, including the evaluation of the in vitro starch digestion.

## 2. Materials and Methods

### 2.1. Raw Materials, Recipes, and Baking Conditions

To obtain triticale malt, native triticale seeds (Etere variety) were put into an automatic Micromalting System (Phoenix Biosystems, Adelaide, South Australia) as described in Gianinetti et al. [17]. The malting process lasted for six days. The durations of the malting cycles with the corresponding temperatures are shown in Table 1.

After the process, rootlets and coleoptiles were removed manually. Native and malted triticale grains were milled (final fineness < 300 μm; Knife Mill Grindomix GM 200, Retsch, Germany) with pre-cutting at a revolution speed of 4000 min^−1^ for 10 s, followed by fine-grinding at a revolution speed of 10,000 min^−1^ for 20 s.

The composite flours for biscuit formulation were prepared by replacing native triticale flour (TF) with malted triticale flour (MTF) at 0, 25, 50, 75, and 100% *w/w*. Biscuits prepared with 100% TF were used as control. Biscuits were made using the following ingredients: 240 g of composite flour, 120 g of whole eggs, 60 g of skimmed milk, 50 g of creamed butter, and 1 g of salt. The ingredients were mixed and manually kneaded for 5 min, and the resulting doughs were kept at 4 °C for 1 h. Then, the doughs were sheeted to a 5 mm thickness with a rolling pin and cut into circular shapes of 3 cm in diameter. Biscuits were baked (180 °C for 15 ± 2 min) in a household oven (RKK 66130, Rex International, Mestrino, Italy). For each recipe, three different biscuit batches were produced.

### 2.2. Physical Characteristics of Doughs and Biscuits

The surface color of the doughs and the biscuits were measured using a Minolta CR410 Chroma Meter (Konica Minolta Co., Tokyo, Japan) based on CIELAB system color space L*, a*, b* values, with reference to the D65 standard illuminant and a visual angle of 10. The parameter L* represents the lightness of the sample, whereas a* (degree of redness) and b* (degree of yellowness) are chromatic components [18,19].

The thickness and diameter of biscuits were evaluated by using a Vernier caliper (on average 20 readings for each thesis). The ratio of diameter/thickness was used to calculate the spread ratio.

### 2.3. Chemical Composition of Samples

The biscuits were finely ground (final fineness < 300 μm; Knife Mill Grindomix GM 200, Retsch, Germany), and the moisture was measured gravimetrically using the air oven method (method 930.15) [20]. The water activity (a_w_) was determined using the water activity meter Aqualab 4TE (Meter Food, Munich, Germany). Ash (method 942.05), crude protein (method 976.05), crude lipid (method 954.02 without acid hydrolysis) total starch (method 996.11), and the content of D-Fructose and D-Glucose (method 985.09) were considered [20]. The total dietary fiber (TDF) content was assessed enzymatically (Megazyme assay kit K-INTDF 02/15; Neogen, Lansing, MI, USA) on flours and biscuits.

### 2.4. Texture Evaluation of Biscuits

A Texture Analyser TexVol TVT 6700 (Perten Instruments, Hägersten, Sweden) with a cylinder probe of 45 mm diameter was used. The instrument was calibrated before the measurements were performed with the following settings: the height of the sample was 15 mm, the probe starting distance was 5 mm, the probe speed was 3 mm/s, the trigger force was 50 g, and the load cell was 5–10 kg. The maximum force recorded during the first compression stroke detected the firmness (g), and the adhesiveness (g) was measured by the work required to overcome the sticky forces between the sample and the probe. The second compression stroke determined the force B (g), the cohesiveness was calculated by the ratio force B/firmness, and the springiness (m) was obtained by the distance of the detected height (firmness) during the second compression divided by the original compression distance [21]. In addition, gumminess (g), conceived as the energy required to disintegrate a semisolid food to make it ready for swallowing (firmness × cohesiveness), and the chewiness (g), the energy needed to chew a solid food until it is ready for swallowing (firmness × cohesiveness × springiness), were measured [21]. For each batch, 15 biscuits were tested.

### 2.5. In Vitro Starch Digestion

A 2-step (i.e., gastric and pancreatic phases) static in vitro starch digestion procedure was used [22]. Briefly, samples were inserted in 50 mL tubes containing glass balls and pre-treated with a 0.05 M HCl solution (5 mL) containing pepsin (5 mg/mL; Sigma P-7000, Sigma-Aldrich^®^ Co., Milan, Italy) for 30 min at 37 °C. The pH was then adjusted to 5.2 by adding 0.1 M sodium acetate buffer before the addition of 5 mL of an enzyme mixture with an amylase activity of about 7000 U/mL given by pancreatin (about 7500 FIP-U/g; Merck 7130, Merck KGaA, Darmstadt, Germany), amyloglucosidase (about 300 U/mL; Sigma A-7095, Sigma-Aldrich^®^ Co., Milan, Italy), and invertase (about 300 U/g; Sigma I-4504, Sigma-Aldrich^®^ Co., Milan, Italy) [22]. Aliquots (0.5 mL) were taken from each tube at 0 (before the addition of the enzyme mixture simulating the pancreatic phase), 30, 60, 120, and 180 min after the addition; absolute ethanol was added, and the amount of released glucose was determined colorimetrically (GODPOD 4058, Giesse Diagnostic snc, Rome, Italy). A blank was also included. A factor of 0.9 was used to convert mono to polysaccharide. The in vitro predicted glycemic index (pGI) was calculated as reported by Giuberti et al. (2015) [22]. For each treatment, samples were analyzed in triplicate.

### 2.6. Statistical Analyses

Data are presented as the mean values ± standard deviation of at least three replicates. The analysis of variance (One-way ANOVA) with a post hoc Tukey test at *p* < 0.05 using IMB SPSS Statistics software (Version 25) was used for data comparison.

## 3. Results

### 3.1. Chemical Composition of Native and Malted Grains

The total starch content of the malted grains was significantly (*p* < 0.05) lower than that of the native grains (Table 2). On the contrary, similar crude protein and ash contents were reported comparing native versus malted grains. The TDF content increased from 11.1 to 17.3 g/100 g of flour comparing native to malted triticale flour, respectively.

### 3.2. Physical Characteristics of Doughs and Biscuits

**Figure 1:**
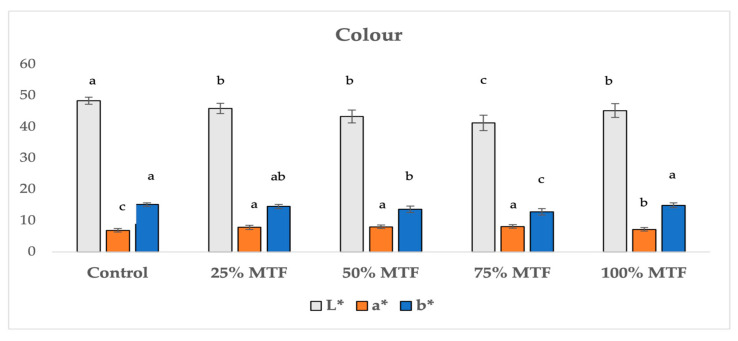
L*, a*, and b* color values of triticale-based biscuits. Control: biscuits prepared with 100% native triticale flour (TF). 25% MTF: biscuits prepared by replacing 25 g/100 g *w/w* of TF with malted triticale flour (MTF). 50% MTF: biscuits prepared by replacing 50 g/100 g *w/w* of TF with MTF. 75% MTF: biscuits prepared by replacing 75 g/100 g *w/w* of MF with MTF. 100 MTF: biscuits prepared with 100% MTF. Different superscript letters in the same parameter are significantly different at the *p* < 0.05 level. The analysis of variance (One-way ANOVA) with a post hoc Tukey test was used. Data are presented as the mean values ± standard deviation of at least three replicates. Details of biscuits prepared with native and malted triticale flours are available in Supplementary Figure S1.

The color of the dough and biscuits was affected (*p* < 0.05) by increasing levels of MTF in the recipe (Table 3 and Figure 1). In doughs, the L* value decreased (*p* < 0.05) from 44.82 (control) to 41.01 (100% MTF), thus indicating a darkening of the products following MTF inclusion. In addition, the b* values showed a similar trend, decreasing from 14.36 (control) to 12.37 (100% MTF), while for the a* values, only the 25% MTF reported a difference (*p* < 0.05) to the control and the other MTF dough samples, showing the highest value (7.16; *p* < 0.05). Similarly, all the MTF-containing biscuits were darker (*p* < 0.05) than the control (i.e., 48.36), with the darkest sample being the 75% MTF (41.31; *p* < 0.05). In addition, control biscuits were characterized by the lowest b* value (*p* < 0.05), whereas the 75% MTF biscuits had the greatest a* values when compared to the other samples (*p* < 0.05) (Figure 1). In addition, color changes, in terms of lower L* and b* values and greater a* values, were obtained by comparing doughs and biscuits at the same inclusion level of MFT in the recipe, due to the baking of the products.

Biscuits were similar in diameter (Table 4), but different (*p* < 0.05) thickness and spread ratio values were recorded among the different formulations. All the MTF-containing samples were characterized by a higher thickness than the control, with the 100% MTF showing the highest value (1.55; *p* < 0.05). Consequently, different spread ratio values were recorded; the spread ratio of all the MTF-containing samples were lower than the control (*p* < 0.05; Table 4).

### 3.3. Chemical Composition of Doughs and Biscuits

Control and MTF-containing doughs reported a similar moisture content (Table 5); only the 100% MTF dough was characterized by a lowest moisture content (*p* < 0.05; 41.2 g water/100 g product). In contrast, the biscuits’ moisture content increased with the addition of MTF in the recipe. Biscuits with 50 and 100% MTF had a higher moisture content compared to the control and to products with lower levels of MTF, being 17.03 g and 18.89 g water/100 g food, respectively (*p* < 0.05). The incorporation of MTF significantly (*p* < 0.05) decreased the a_w_ of doughs (Table 4) and biscuits (Table 5). In addition, the total starch content of biscuits decreased (*p* < 0.05) as the inclusion level of MTF increased in the recipe, ranging from 49.51% to 36.15% of dry matter, respectively, for the control and 100% MTF biscuits. The glucose and fructose content (*p* < 0.05) increased from 0.31% (control) to 2.80% (100% MTF) and from 0.24% (control) to 1.29% g/100 d dry matter (100% MTF), respectively. Similar results (*p* < 0.05) were reported considering the crude protein content of samples. With regards to the ash content, 75 and 100% MTF biscuits showed the highest values (*p* < 0.05), having 1.61–1.62% dry matter. The TDF of biscuits increased (*p* < 0.05) as the substitution levels of MTF increased from 25 to 100% *w/w*. In particular, 100% MTF biscuits approximately reached values that were three times higher than the value of the control sample. The dietary fiber content ranged from 3.49% to 9.92% dry matter for the control and biscuits formulated with 100% MTF, respectively. 

The in vitro starch hydrolysis index (starch HI) and the predicted glycemic index (pGI) (*p* < 0.05) increased with increasing levels of MTF in the formulation. The highest starch HI and pGI occurred in 100% MTF biscuits, reaching 104.43 and 98.22, respectively, while the control and the 25% MTF showed the lowest results: 59.50 and 62.51 for the starch HI and 59.49 and 62.08 for the pGI value, respectively (Table 5).

### 3.4. Texture Evaluation of Biscuits

Increasing the level of MTF in the biscuit recipe contributed to modifying the texture of the final products (Table 6). In particular, firmness, force B, chewiness, and gumminess decreased (*p* < 0.05) with increasing levels of MTF in the recipe, with the lowest values being recorded for 100% MTF samples (*p* < 0.05). The 25% MTF biscuits showed similar values to the control in firmness, chewiness, and gumminess; however, they reported significantly (*p* < 0.05) higher force B than the control and the highest springiness value (*p* < 0.05). Similar cohesiveness values were reported among the different formulations.

## 4. Discussion

Nowadays, the interest in formulating baked products with under-exploited flour is increasing, driven by consumers’ demand for healthier food products [1]. In this regard, germination has been identified as an inexpensive and effective green technology to improve the quality of cereal and legume grains by enhancing nutrient content and digestibility and reducing the levels of antinutrients [14,15,19,23,24,25,26,27]. The effect of germination on nutrient contents has been widely studied; however, very little information is found in the literature about the effects of germination/malting on triticale composition and physicochemical properties necessary to know possible food applications [1,19].

The MTF incorporation into the biscuit recipe contributed to changes in several physio–chemical characteristics. As expected, the increase in MTF level caused doughs and biscuits to darken and brown, in line with previous findings [28]. This mainly occurs because of the increase in small molecules produced by the enzymatic degradation of starch and protein during germination. The small molecules primarily involved are reducing sugars and amino acids, which, during baking, can react, originating the Maillard reaction, a range of reactions that lead to the formation of brown nitrogenous polymers and co-polymers known as melanoidins [28,29,30]. In addition, the color changes in biscuits can also be attributed to the caramelization of reducing sugars during cooking [29,31].

The diameter of biscuits was not influenced by the addition of MTF. However, as MTF substitution level increased in the recipe, the thickness increased, probably due to the more intense indigenous yeast activity in the presence of free sugars [30]. Consequently, biscuits with higher MTF amounts obtained lower spread ratio values. The biscuit spread ratio represents the ratio of diameter to height. Thus, the effects of free sugars on the diameter (sugar dissolution) and height (inhibiting gluten development) are combined into a single parameter [27]. During malting, enzymatic degradation of starch and protein in flours to smaller sugars and peptides may occur. As a result, the hydrophilic nature of the biscuits can be increased, thus contributing to the decrease in the spread factor. Higher spread ratio values are considered an important quality attribute of biscuits because of their relationship with texture, bite, and overall mouthfeel [27]. The lowering in the spread ratio value can also be related to MTF containing more water-absorbing constituents like fiber and protein, as already reported [27,28,29]. Comparable results were reported in biscuits containing different malted flours [28,29].

Data indicated that the MTF inclusion in the formulation increased the moisture content of products. Overall, dry biscuits should have a moisture content lower than 5 g/100 g of product after baking and generally an a_w_ of 0.4 [27]. The substitution of native triticale flour with increasing levels of MTF increased biscuits’ moisture content in the study of Chung et al. [32]. In addition, Karimzadeghan et al. [33] observed that the moisture content of the samples containing triticale significantly increased due to the high mineral and fiber content of triticale flour compared to wheat flour. On the contrary, the decrease in a_w_ following MTF increasing inclusion levels might be related to the binding of water to smaller molecules broken by enzymes during germination [15,30]. However, considering both the relatively high moisture and a_w_ levels in the experimental biscuits, shelf-life studies are strongly warranted to evaluate the microbiological stability of the newly developed products. In addition, different baking conditions should be tested, aiming to reduce moisture and a_w_ levels in these products.

Biscuits with increasing levels of MTF presented enhanced nutritional characteristics, owing, in part, to the increase in TDF and protein and to the decrease in total starch content. Several studies reported increased TDF in different germinated cereals at different germination times and temperatures [15,19,28,32]. This increase could be due to the solubilization of the relevant macromolecules, the cleavage of intermolecular bonds, and the breakdown of protein structures [34]. The increase in TDF can partly be explained by the loss of compounds such as starch due to respiration and the synthesis of new polysaccharides during germination, which can cause changes in the cell wall matrix [15,28,30,34]. In terms of TDF, biscuits may therefore be ordered as follows: 100% MTF > 75% MTF > 50% MTF > 25% MTF > control. Dietary fiber exerts several benefits to human health and wellbeing. Plenty of studies have suggested that higher consumption of dietary fiber is beneficial for a variety of health outcomes, including, but not limited to, the prevention of arteriosclerosis, protection against colon cancer, lower concentrations of serum inflammatory biomarkers, and a lower risk of coronary heart disease [1,8,14,19]. Consequently, the use of MTF could be considered a valuable strategy to formulate baked goods that might promote a higher fiber intake as part of a healthy diet.

Ash content significantly increased starting from 75% MTF; this measure is an indication of the mineral’s constituents present in the food. Several authors observed increased mineral content (Fe, Zn, Ca, Se) after grain germination [28,30,34]. This occurs for the activation of phytase, which hydrolyses phytic acid during germination, making minerals more bioavailable [15,30].

Adding MTF to native triticale flour in the biscuit recipe resulted in a slightly higher protein content of all MTF-containing biscuits to the control, with no differences among the different inclusion levels. This suggests that MTF could contain small amounts of amino acids synthesized during germination, which were added to the intact proteins of native flour, thus providing a higher protein content. Analogously, Chauhan et al. [35] observed increased protein content in germinated amaranth flour, and Aluge et al. [36] found that protein content increased with increasing malted sorghum flour substitution. Authors indicated that the increase in protein content in germinated/malted flours could be related to the synthesis of enzymes, which might have resulted in the production of some amino acids during protein synthesis.

As expected, the use of MTF determined a decrease in total starch and an increase in reducing sugars in triticale-based biscuits. According to Baranzelli et al. [37], 24 h for germination is insufficient to activate the amylolytic enzymes because their maximum hydrolysis activity is between 48 and 72 h. In this study, a total of 96 h of germination was employed. Our findings agree with previous findings, in which glucose and fructose contents increased considerably throughout the malting process [15,19,28,32].

Food with low starch HI and pGI values would promote slow and moderate postprandial glucose and insulin responses; thus, these foods can be more desirable for diabetic patients as well as for healthy individuals [38]. However, results obtained from the in vitro digestion indicated that the addition of higher levels of MTF in biscuits determined an increase in the starch HI and the pGI, and hence an enhancement of the overall in vitro starch digestibility. This may be somewhat undesirable, since current nutritional guidelines encourage the consumption of carbohydrate-rich foods with slowly digestible starch properties to promote good health [38]. The increase in HI and pGI following MTF inclusion in biscuit recipe can be attributed to the lower total starch content in MTF biscuits and the higher content of glucose. The present findings agree with the study of Yang et al. [34], showing that germination enhanced the starch digestibility of different cereal flours. A downside of germination can be that the inherent starch structure is degraded by the action of the enzyme hydrolysis, making it easier for starch to be degraded by amylase enzymes [15]. In addition, germination can also promote the activity of α-amylase and deactivate some amylase inhibitors [15,34]. The pGI is based on the in vitro release of glucose following carbohydrate hydrolysis. Hence, it is strongly linked with the content of glucose and starch morphology. In the present study, biscuits with MTF replacement above 25% obtained high pGI indices (pGI > 70), whereas the control and biscuits with 25% MTF showed medium values (56 < pGI < 69) [38]. On the contrary, Molinari et al. [29] showed that malted tartary cookies presented lower HI and pGI compared to native tartary flour cookies because of the higher content of dietary fiber and resistant starch. Even Cornejo et al. [39] found that the HI as well as the pGI of bread were significantly reduced with the germination of brown rice. Present in vitro results indicated that the structural attributes of products (i.e., hardness) can also contribute to dictate the in vitro breakdown pattern of starch. It has been widely reported that samples with similar composition may also be digested in vitro at different rates and extents depending on their structural attributes [22,30,39]. However, discrepancies among studies can be related to the different chemical composition of products; the different germination, malting, and baking conditions employed; and differences in the in vitro systems used. This suggests the need for harmonization of the in vitro starch digestion systems to make data from different studies more comparable. In addition, the enhanced in vitro starch digestibility of MTF-containing biscuits should be carefully considered to optimize the use of MTF in biscuit formulation. Nevertheless, these results based on in vitro studies clearly warrant further in vivo studies.

Regarding texture results, MTF-containing biscuits became softer with the increasing substitution of MTF. Similar results were obtained by Chung et al. [32] for cookies containing germinated brown rice flour and by Chauhan et al. [35] for cookies made with germinated amaranth flour. The decrease in hardness could be attributed to the formation of a weaker matrix in biscuits caused by the structural degradation of protein and starch during germination. In addition, this decrease might be related to the increasing dietary fiber content following the incorporation of MTF, as well as to the higher level of water in the products. Sozer et al. [40] observed that the cookie’s firmness decreased with the highest contents of fiber incorporation. Because of the decrease in firmness, biscuits with the highest MTF levels showed a reduction in the second compression (force B), in gumminess, in chewiness, and an increase in springiness. However, the latter parameter did not increase linearly with MTF inclusion levels, as 25% of MTF in biscuit formulation obtained the highest value, while 75% of MTF resulted in the lowest value. All the samples showed higher springiness values compared with the optimal values reported in the literature, showing that the optimal range is in the range of 0.05–0.72 [27].

## 5. Conclusions

This study reveals that triticale and malted triticale flours can be used in biscuit formulation. Increasing the incorporation of malted triticale flour contributed to enhancing the nutritional value of triticale-based products by mainly increasing the fiber content. From a nutritional standpoint, starting from the threshold of 50% of malted triticale flour substitution, biscuits could benefit from the claim of “high fiber content”, with the total dietary fiber content being >6 g/100 g [41]. Changes occurred considering the technological parameters since a decrease in firmness, chewiness, gumminess, and spread ratio was reported. However, the lowering in these texture values can be tailored for specific consumers who have chewing problems, such as the elderly population. Considering the in vitro starch digestion, chemical modifications following the malting process of triticale flour influenced the in vitro digestibility of starch by increasing the in vitro starch hydrolysis index. This aspect should be carefully considered and better investigated through in vivo trials. Data indicated that substituting native with malted triticale flour could contribute to formulating food products with attractive nutritional properties without drastically influencing technological parameters. However, further in vivo studies and a shelf-life evaluation are needed to better explore the potential of native and malted triticale flour in biscuit food formulation. In addition, since malting can promote the development of more desirable aromas and flavors, a sensory evaluation of products is warranted. Lastly, future studies are needed to optimize the malting protocol to obtain the highest total dietary fiber or protein contents to improve the nutritional profile of malted triticale flour for different food applications.

## Figures and Tables

**Table 1:** Malting stages, duration, initial temperature (Ti), and final temperature (Tf), are expressed as °C.

Malting Stage	Hours	Minutes	Ti	Tf
Cleaning		15		
Steeping 1	7	15	15	15
Germination 1	8		19	19
Steeping 2	9		15	15
Germination 2	6		19	19
Steeping 3		30	15	15
Germination 3	39	30	15	15
Germination 4	24		15	15
Germination 5	25		15	15
Kilning 1	7		30	40
Kilning 2	6		40	60
Kilning 3	6	30	60	70
Kilning 4	4	30	70	80
Kilning 5		30	25	25

**Table 2:** Chemical composition of native and malted Etere triticale flours (g/100 g flours).

Triticale	Crude Protein	Total Starch	Ash	TDF
Native	12.3 ± 0.49 ^a^	58.4 ± 4.04 ^a^	2.6 ± 0.02 ^a^	11.1 ± 2.21 ^b^
Malted	13.7 ± 0.18 ^a^	52.1 ± 1.92 ^b^	1.7 ± 0.01 ^a^	18.3 ± 3.01 ^a^

Mean values with different superscripts within column are significantly different at (*p* < 0.05). Data are presented as the mean values ± standard deviation of at least three replicates. TDF: total dietary fiber.

**Table 3:** Physical and chemical characteristics of triticale-based doughs.

	Control	25% MTF	50% MTF	75% MTF	100% MTF
L*	44.82 ± 0.63 ^a^	45.43 ± 0.75 ^a^	43.22 ± 0.93 ^b^	42.38 ± 0.89 ^b^	41.01 ± 1.02 ^c^
a*	6.66 ± 0.25 ^b^	7.16 ± 0.29 ^a^	6.93 ± 0.24 ^b^	6.81 ± 0.19 ^b^	6.60 ± 0.18 ^b^
b*	14.36 ± 0.62 ^a^	14.04 ± 0.48 ^a^	13.37 ± 0.51 ^b^	13.38 ± 0.45 ^b^	12.37 ± 0.63 ^c^
Moisture ^1^	44.76 ± 0.01 ^a^	43.78 ± 0.01 ^a^	44.68 ± 0.02 ^a^	44.99 ± 0.02 ^a^	41.22 ± 0.02 ^b^
a_w_	0.973 ± 0.002 ^a^	0.959± 0.001 ^b^	0.946 ± 0.004 ^b^	0.944 ± 0.001 ^b^	0.925 ± 0.005 ^c^

Control: dough prepared with native 100% triticale flour (TF). 25% MTF: dough prepared by replacing 25 g/100 g *w/w* of TF with malted triticale flour (MTF). 50% MTF: dough prepared by replacing 50 g/100 g *w/w* of TF with MTF. 75% MTF: dough prepared by replacing 75 g/100 g *w/w* of MF with MTF. 100 MTF: dough prepared with 100% MTF. Mean values with different superscripts within rows significantly differ (*p* < 0.05). The analysis of variance (One-way ANOVA) with a post hoc Tukey test was used. Data are presented as the mean values ± standard deviation of at least three replicates. ^1^ g water/100 g food.

**Table 4:** Physical characteristics of triticale-based biscuits.

	Control	25% MTF	50% MTF	75% MTF	100% MTF
Diameter (mm)	3.28 ± 0.10 ^a^	3.45 ± 0.09 ^a^	3.47 ± 0.08 ^a^	3.56 ± 0.06 ^a^	3.54 ± 0.07 ^a^
Thickness (mm)	1.38 ± 0.11 ^c^	1.47 ± 0.11 ^b^	1.51 ± 0.08 ^b^	1.48 ± 0.11 ^b^	1.55 ± 0.16 ^a^
Spread ratio (D/T)	2.46 ± 0.20 ^a^	2.36 ± 0.19 ^b^	2.29 ± 0.12 ^c^	2.39 ± 0.17 ^b^	2.31 ± 0.21 ^c^

Control: biscuits prepared with native 100% triticale flour (TF). 25% MTF: biscuits prepared by replacing 25 g/100 g *w/w* of TF with malted triticale flour (MTF). 50% MTF: biscuits prepared by replacing 50 g/100 g *w/w* of TF with MTF. 75% MTF: biscuits prepared by replacing 75 g/100 g *w/w* of MF with MTF. 100 MTF: biscuits prepared with 100% MTF. Mean values with different superscripts within rows are significantly different at (*p* < 0.05). The analysis of variance (One-way ANOVA) with a post hoc Tukey test was used. Data are presented as the mean values ± standard deviation of at least three replicates.

**Table 5:** Chemical composition (g/100 g dry matter), in vitro starch hydrolysis index (HI), and in vitro predicted glycemic index (pGI) of biscuits.

	Control	25% MTF	50% MTF	75% MTF	100% MTF
Moisture ^1^	14.22 ± 0.02 ^c^	14.23 ± 0.01 ^c^	18.35 ± 0.04 ^a^	17.03 ± 0.00 ^b^	18.89 ± 0.04 ^a^
a_w_	0.859 ± 0.012 ^a^	0.859 ± 0.006 ^a^	0.806 ± 0.012 ^b^	0.801 ± 0.022 ^b^	0.759 ± 0.003 ^c^
Crude protein	12.44 ± 0.47 ^b^	13.09 ± 1.02 ^a^	13.56 ± 0.37 ^a^	13.53 ± 0.58 ^a^	13.84 ± 0.42 ^a^
Crude lipid ^ns^	16.72 ± 0.06	16.11 ± 0.17	16.47 ± 0.15	16.53 ± 0.02	15.74 ± 0.05
Total starch	49.51 ± 1.64 ^a^	46.52 ± 1.25 ^b^	40.45 ± 1.94 ^c^	37.28 ± 2.14 ^d^	36.15 ± 1.87 ^d^
Glucose	0.31 ± 0.00 ^d^	1.05 ± 0.01 ^c^	1.23 ± 0.02 ^c^	1.72 ± 0.03 ^b^	2.80 ± 0.07 ^a^
Fructose	0.24 ± 0.00 ^d^	0.51 ± 0.00 ^c^	0.59 ± 0.01 ^c^	0.87 ± 0.01 ^b^	1.29 ± 0.01 ^a^
Ash	1.59 ± 0.07 ^b^	1.59 ± 0.00 ^b^	1.60 ± 0.01 ^ab^	1.61 ± 0.00 ^a^	1.62 ± 0.00 ^a^
Total dietary fiber	3.49 ± 0.57 ^e^	5.09 ± 1.11 ^d^	7.21 ± 0.98 ^c^	9.54 ± 1.02 ^b^	9.92 ± 1.16 ^a^
Starch HI ^2^	59.50 ± 2.56 ^d^	62.51 ± 2.45 ^d^	78.89 ± 2.04 ^c^	88.31 ± 3.01 ^b^	104.43 ± 3.33 ^a^
pGI	59.49 ± 2.99 ^d^	62.08 ± 2.54 ^d^	76.20 ± 2.33 ^c^	84.32 ± 1.44 ^b^	98.22 ± 2.23 ^a^

Control: biscuits prepared with 100% native triticale flour (TF). 25% MTF: biscuits prepared by replacing 25 g/100 g *w/w* of TF with MTF. 50% MTF: biscuits prepared by replacing 50 g/100 g of TF with MTF. 75% MTF: biscuits prepared by replacing 75 g/100 g of MF with MTF. 100 MTF: biscuits prepared with 100% malted triticale flour (MTF). Mean values with different superscripts within lines are significantly different at (*p* < 0.05). ns superscript within one line denotes means were not significantly different (*p* ≥ 0.05). The analysis of variance (One-way ANOVA) with a post hoc Tukey test was used. Data are presented as the mean values ± standard deviation of at least three replicates. ^1^ g water/100 g food. ^2^ Calculated using white wheat bread as reference (HI = 100 by definition) [20].

**Table 6:** Texture evaluation of triticale-based biscuits. All values are reported in grams (g).

	Control	25% MTF	50% MTF	75% MTF	100% MTF
Firmness	21,483.87 ± 3987.87 ^a^	20,913.87 ± 2119.96 ^a^	16,309.29 ± 1991.21 ^b^	16,068.56 ± 1863.12 ^b^	15,543.27 ± 1835.50 ^c^
Force B	18,700.20 ± 3606.08 ^a^	17,995.66 ± 1876.98 ^b^	13,899.94 ± 1695.89 ^c^	13,611.72 ± 1591.88 ^c^	13,337.45 ± 1596.17 ^c^
Springiness	0.89 ± 0.16 ^a^	0.97 ± 0.06 ^c^	0.91 ± 0.11 ^b^	0.87 ± 0.14 ^a^	0.94 ± 0.08 ^b^
Cohesiveness ^ns^	0.5 ± 0.04 ^a^	0.5 ± 0.02 ^a^	0.5 ± 0.01 ^a^	0.5 ± 0.01 ^a^	0.5 ± 0.03 ^a^
Chewiness	10,741.60 ± 2458.83 ^a^	10,672.80 ± 1193.46 ^a^	8010.70 ± 1142.59 ^b^	7965.28 ± 1112.44 ^b^	7921.18 ± 1147.62 ^c^
Gumminess	10,750.13 ± 2460.51 ^a^	10,671.20 ± 1192.92 ^a^	8008.00 ± 1138.10 ^b^	7962.56 ± 1111.79 ^b^	7914.36 ± 1146.52 ^b^

Control: biscuits prepared with 100% native triticale flour (TF). 25% MTF: biscuits prepared by replacing 25 g/100 g *w/w* of TF with MTF. 50% MTF: biscuits prepared by replacing 50 g/100 g of TF with MTF. 75% MTF: biscuits prepared by replacing 75 g/100 g of MF with MTF. 100 MTF: biscuits prepared with 100% malted triticale flour (MTF). Mean values with different superscripts within rows are significantly different at (*p* < 0.05). ns superscript within one row denotes means were not significantly different (*p* ≥ 0.05). The analysis of variance (One-way ANOVA) with a post hoc Tukey test was used. Data are presented as the mean values ± standard deviation of at least three replicates.

## Data Availability

The datasets generated for this study are available on request to the corresponding author.

## Supplementary Materials

The following supporting information can be downloaded at: https://www.mdpi.com/article/10.3390/foods12183418/s1, Figure S1: Details of biscuits prepared with native and malted triticale flours.
