# Supplementary material for: Combining Native and Malted Triticale Flours in Biscuits: Nutritional and Technological Implications
Source: Foods. 2023 Sep 13;12(18):3418. doi: 10.3390/foods12183418 (PMC10529920; doi:10.3390/foods12183418)
Supplement: Supplementary file 1 [file foods-12-03418-s001.zip › foods-2552016-supplementary.pdf]

Supplementary Figure S1. Details of biscuits prepared with native and malted triticale flours.

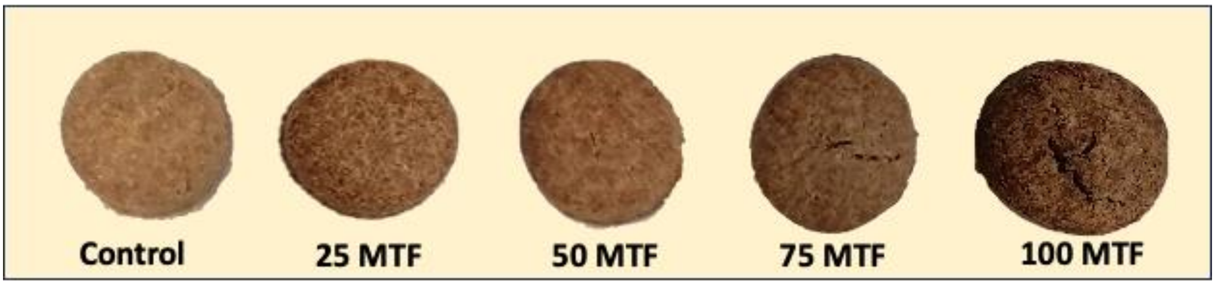

Control: biscuits prepared with 100% native triticale flour (TF). 25% MTF: biscuits prepared by replacing 25g/100g w/w of TF with malted triticale flour (MTF). 50% MTF: biscuits prepared by replacing 50g/100g w/w of TF with MTF. 75% MTF: biscuits prepared by replacing 75g/100g w/w of MF with MTF. 100 MTF: biscuits prepared with 100% MTF.
